# How are Informal Caregivers Adapting to COVID19? Preliminary Results of an Online Survey

**DOI:** 10.1093/geroni/igaa057.3471

**Published:** 2020-12-16

**Authors:** Ranak Trivedi, Madhuvanthi Suresh, Rashmi Risbud, Marika Humber, Josephine Jacobs, Samuel Thomas

**Affiliations:** 1 VA Palo Alto/Stanford University, Menlo Park, California, United States; 2 Georgia Regional Hospital-Atlanta, Atlanta, Georgia, United States; 3 VA Palo Alto Health Care System, Palo Alto, California, United States; 4 VA Palo Alto Health Care System, Menlo Park, California, United States; 5 Palo Alto Veterans Health Administration, Menlo Park, California, United States; 6 Stanford University School of Medicine, Salt Lake City, Utah, United States

## Abstract

COVID19 may disrupt informal caregivers’ (CG) ability to support their care recipients (CR) but little is known how caregivers adapt. A 10-minute, anonymous, online survey with no geographic restrictions was fielded April-August 2020. Two screening items ensured that the respondents were at least 18y and self-identified as a CG. This English-language survey assessed: sociodemographics; reactions to COVID19; changes in CG responsibilities and abilities; depression (Patient Health Questionnaire, PHQ-2); CG burden (Zarit Burden Inventory, ZBI-4); and anxiety (Generalized Anxiety Disorder, GAD-2). Univariate analyses determined the proportion of those who screened positive on PHQ-2 (cutoff=3), GAD-2 (cutoff=3), and ZBI-4 (cutoff=8). Of the 314 respondents, 74% lived in USA; 73.5% of caregivers and 48.2% CR were women. While 63.4% were married, only 28% cared for their spouse. CG mainly cared for adults (83%), and reported that 75.0% of their CR had 2+ conditions. 49.6% CG provided >20h of care/wk. Since COVID19, 53% reported an increase in CG responsibilities; 28.0% noted a decrease in income. Many CG screened positive on the ZBI-4 (48.4%), GAD-2 (30.9%), and PHQ-2 (26.8%). 74% worried about contracting COVID19 at least some of the time. 35.0% noted limits to performing all caregiving tasks when they (N=34) or their CR (N=57) were asked to self-isolate/quarantine. 163 (51.9%) CG noted spending less time with their CR, of which 46.4% used alternate means (e.g., telephone calls). Preliminary results show that a plurality of CG had changes in their responsibilities and abilities during COVID19. A sizable proportion also reported poor well-being.

